# 1321. Acquisition and Transmission of *Streptococcus pneumoniae* in Individuals Over the Age of 60 Years Residing in New Haven, CT, USA

**DOI:** 10.1093/ofid/ofab466.1513

**Published:** 2021-12-04

**Authors:** Anne Wyllie, Sidiya Mbodj, Devyn Yolda-Carr, Darani A Thammavongsa, Pari Waghela, Maura Nakahata, Noel J Vega, Anna York, Orchid M Allicock, Ronika Alexander-Parrish, Adriano Arguedas, Bradford D Gessner, Daniel Weinberger, Maikel Hislop

**Affiliations:** 1 Yale School of Medicine, New Haven, Connecticut; 2 Yale School of Public Health, New Haven, Connecticut; 3 Yale University, New Haven, Connecticut; 4 Pfizer, Inc, Bowie, MD; 5 Pfizer Inc, Collegeville, Pennsylvania; 6 Pfizer Vaccines, Collegeville, PA

## Abstract

**Background:**

Despite the widespread use of pneumococcal conjugate vaccines, particularly in children, an important burden of pneumococcal disease remains in older adults. The acquisition and transmission rates of pneumococcus between older adults have not been well characterized.

**Methods:**

Between October 2020-June 2021, couples living in the Greater New Haven Area were enrolled if both individuals were over the age of 60 years and did not have any individuals under the age of 60 years living in the household. Saliva samples and questionnaires regarding social patterns and medical history were obtained every 2 weeks for a period of 10 weeks. Following culture-enrichment, extracted DNA was tested using qPCR for pneumococcus-specific sequences *piaB* and *lytA*. Individuals were considered positive for pneumococcal carriage when qPCR Ct-values for *piaB* +/- *lytA* were less than 40.

**Results:**

To date, we have collected 495 saliva samples from 95 individuals (48 households). Of 495 saliva samples, 31 (5.9%) have tested positive for pneumococcus by either *piaB* only (n=9) or both *lytA* and *piaB* (n=22). Of 95 individuals, 16 (16.8%) (representing 13, or 27.1% households) have tested positive at least once. Six of the 16 (37.5%) carriers tested positive at multiple timepoints, though none were colonized at all 6 time points over the course of the 10 weeks of study enrolment. For 3 of the 48 (6.3%) households, both members of the couple were identified as carriers, though not necessarily at the same sampling moment.

**Conclusion:**

The preliminary findings of this longitudinal transmission model demonstrate evidence of pneumococcal acquisition among older adults measured by molecular tools. These transmission patterns and high rates of pneumococcal carriage in adults were observed during a period when the COVID-19 pandemic led to numerous preventative public health measures that may have reduced pneumococcal transmission (e.g., social distancing, mask wearing, bans on mass gatherings, restaurant closures, travel restrictions).

**Disclosures:**

**Anne Wyllie, PhD**, **Global Diagnostic Systems** (Consultant)**Pfizer** (Advisor or Review Panel member, Research Grant or Support)**PPS Health** (Consultant)**Tempus Labs, Inc** (Research Grant or Support) **Ronika Alexander-Parrish, RN, MAEd**, **Pfizer** (Employee, Shareholder) **Adriano Arguedas, MD**, **Pfizer** (Employee) **Bradford D. Gessner, MD, MPH**, **Pfizer Inc.** (Employee) **Daniel Weinberger, PhD**, **Affinivax** (Consultant)**Merck** (Consultant, Grant/Research Support)**Pfizer** (Consultant, Grant/Research Support)

